# Dynamic Risk Stratification for Predicting Treatment Response in Differentiated Thyroid Cancer

**DOI:** 10.3390/jcm9092708

**Published:** 2020-08-21

**Authors:** Evanthia Giannoula, Christos Melidis, Nikitas Papadopoulos, Panagiotis Bamidis, Vasilios Raftopoulos, Ioannis Iakovou

**Affiliations:** 1 Second Academic Nuclear Medicine Department, Academic General Hospital of Thessaloniki “AHEPA”, Aristotle University of Thessaloniki, Greece, Kiriakidi 1 St, 54621 Thessaloniki, Greece; iiakovou@auth.gr; 2 CAP Santé, Radiation Therapy Department, 13 Rue Marcel Paul, 20200 Bastia, France; melichristos@hotmail.com; 3 General Hospital of Thessaloniki “Georgios Gennimatas”, Ethnikis Aminis 41 St, 54635 Thessaloniki, Greece; n_papadopoulos@outlook.com; 4 Medical Physics Laboratory, Medical School, Aristotle University of Thessaloniki, 54635 Thessaloniki, Greece; bamidis@med.auth.gr; 5 Division of HIV/AIDS Epidemiological Surveillance, National Public Health Organization (E.O.D.Y.), Agrafon 3–5 St, 15123 Athens, Greece; vraftop1@gmail.com

**Keywords:** thyroid cancer, dynamic risk stratification, treatment response, prognostic factors

## Abstract

Prognosis in Differentiated Thyroid Cancer (DTC) patients is excellent, but a significant degree of overtreatment still exists because of the inability to accurately identify small patient cohorts who experience a more aggressive form of the disease, often associated with certain poor prognostic factors. Identifying these cohorts at an early stage would allow patients at high risk to receive more aggressive treatment while avoiding unnecessary and invasive treatments in those at low risk. Most risk stratification systems include age, tumor size, grade, presence of local invasion, and regional or distant metastases. Here we discuss these common factors as well as their association with treatment response, but also other upcoming markers including histology and multifocality of primary tumor, dose administered and preparation method for Radioiodine Therapy (RAI), Thyroglobulin (Tg), Anti-thyroglobulin Antibodies (Tg-Ab) levels both at initial management and during follow-up, and the presence of previously existing benign thyroid disease. In addition, we examine the role of remnant size and avidity as well as surgeons’ experience in performing thyroidectomies with recurrence rate, discussing its impact on disease prognosis. Our results reveal that treatment response has a statistically significant association with histology, T and M stages, surgeons’ experience, Tg levels and remnant score both during RAI and follow up and Tg-Ab levels during follow-up whole body scan (WBS).

## 1. Introduction

Thyroid cancer is the most common malignant disease of the endocrine system and one of the few cancers with rising incidence [1,2,3]. Differentiated thyroid cancer (DTC) accounts for around 90% of all thyroid tumors, and includes both papillary (PTC) and follicular carcinomas (FTC) [4]. DTC prognosis is generally very good while its outcome depends on the stage of the disease at the time of onset as well as factors such as age, grade, size, extension, distant metastasis, and low- versus high-risk groups for recurrence. However, there are small cohorts of patients who experience a more aggressive form of the disease, often associated with certain poor prognostic factors. In particular, 80% of patients will do well with minimal surgical treatment, while 5% may die regardless of treatment provided. The remaining 15% represents a group of patients who may benefit from a more aggressive oncological resection, with adjuvant radioactive iodine treatment and, in selected cases, external-beam radiation therapy [5]. It is, thus, very important that the treating physician understands the prognostic factors to facilitate individual patient risk group categorization.

Dynamic Risk Stratification (DRS), an active process used to predict the appropriateness for minimalistic initial therapy, disease-specific mortality, risk of recurrence, and the most likely response to initial therapy, is nowadays used in order to properly assess the risk of DTC relapse. Rather than being a static prediction available only after initial therapy, it is a dynamic, iterative process that begins as soon as a suspicious nodule is detected and continues through to final follow-up [6].

The aim of the current retrospective study is to investigate the factors influencing treatment response in DTC patients. Our intention was not only to statistically estimate treatment response based on a small set of clinicopathological features, but rather to perform DRS associating all the aforementioned characteristics from initial diagnosis to follow up, with response to the treatment. In this context, we investigated the impact of demographic, clinical, pathologic, treatment administered and follow-up characteristics, as well as the association of laboratory and imaging results from initial postsurgical management to treatment and follow-up with treatment response.

## 2. Materials and Methods

Demographic, clinical, pathological, diagnostic, treatment, and follow up data were recorded retrospectively for 549 DTC patients who had been consecutively recruited. They had undergone (near) total thyroidectomy with or without lymph node neck dissection, and radioiodine RAI (RAI) procedure or follow up with whole body scintigraphy (WBS) 6–12 months after RAI, in order to accomplish DRS for the cohort. A formal protocol of the study was submitted and approved by the ethics committee of Aristotle University of Thessaloniki, Greece (ethical approval code: 398/11.12.2017). Moreover, before radioiodine treatment, patients provided written informed consent allowing the use of their pseudonymized data in a statistically aggregate manner. Patient recruitment took place at the tertiary referral centers for DTC in Northern Greece, Papageorgiou Hospital of Thessaloniki (PGH) and Cancer Hospital of Thessaloniki (CHT).

The histology report and the American Joint Committee on Cancer TNM system were used to stage each patient’s disease pre-RAI. Response to treatment (RTT) was evaluated according to the latest American Thyroid Association Guidelines (ATA GLs) [7], while a scoring system concerning the referral surgeon’s apparent experience in thyroid surgery and thyroid remnants was used as follows:

Surgeons referring less than 5 patients, between 5 and 9 patients, between 10 and 19 patients or equal to or more than 20 patients received a score of 1, 2, 3, or 4 respectively.

**Figure 1:**
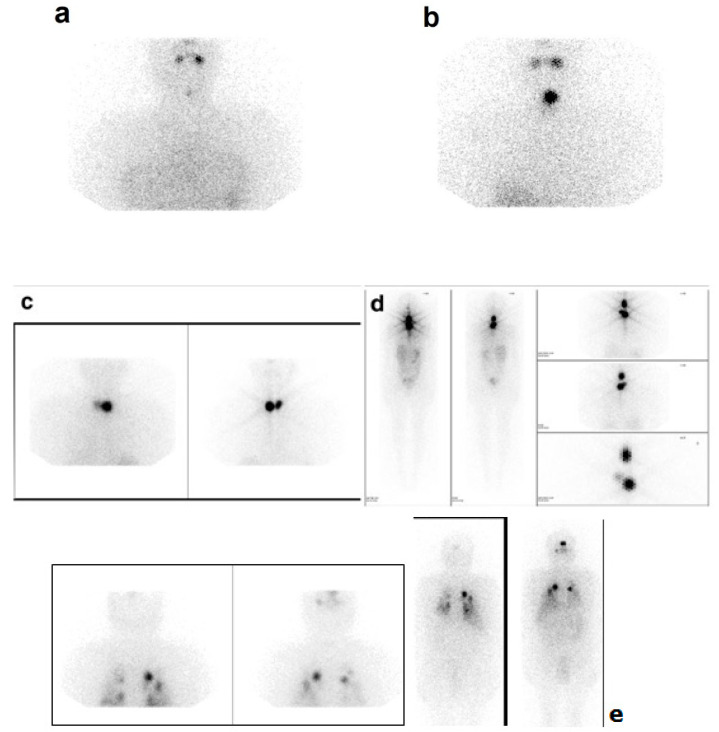
Representative post-ablation whole body scan (WBS) images illustrating scores for intensity of uptake and maximum remnant scores: (**a**) score 1 (low uptake), (**b**) score 2 (intermediate uptake), (**c**) score 3 (high uptake), (**d**) score 4 (star effect), (**e**) score 5 (distant metastases).

Thyroid remnant was classified from 0 to 20 as the product of the number of objectively observed thyroid bed foci times the subjective overall uptake intensity. The following scores were used for foci: 0 foci, 1 point; 1 focus, 2 points; 2 foci, 3 points; >3 foci, 4 points; and for uptake: no uptake, 0 points; low uptake, 1 point; intermediate uptake, 2 points; high uptake, 3 points; star effect, 4 points; distant metastases, 5 points, as shown in Figure 1. Remnant classification scoring was performed by a pair of experienced nuclear medicine physicians at the respective institutions. Each pair worked jointly and disagreements were resolved by consensus.

Post-therapy WBS was performed 3–7 days post-ablative radioiodine administration. Anterior and posterior planar images of the cranium, thorax, and abdomen from the top of the head to the inguinal region, and as warranted, spot images, were obtained while patients were supine. Scanning was performed for ≥500,000 counts.

Tg and Tg-Ab were measured using commercial assays (see Annex Tables S1 and S2 for methodological details) by one accredited central laboratory for each institution. Thyroglobulin (Tg), anti-thyroglobulin autoantibodies (Tg-Ab), and anti-thyroperoxidase autoantibodies (Anti-TPO) were measured by the accredited central laboratories of the recruiting hospitals. Methods used did not change over the period of study. Samples were drawn immediately before radioiodine administration or before WBS in patients undergoing thyroid hormone withdrawal (THW: no thyroid hormone for the 2–6 weeks before radioiodine therapy) or 72 h after the second rhTSH injection, i.e., 48 h post-ablative administration, in those receiving rhTSH.

Each cohort patient was classified in one out of four treatment response categories (according to the latest ATA GLs) based on biochemical and imaging results of the first follow-up WBS performed after TSH stimulation, 6–12 months after RAI [7].

Discrete variables are expressed as counts and percentages, or vice versa, and continuous variables as mean (±SD) or median [IQR]. A chi-square test was used to evaluate the association between categorical variables. A one-way ANOVA (with Fisher’s LSD adjustment for multiple comparisons) test was used to examine the difference of continuous variables between the categories of response. *p* ≤ 0.05 was considered statistically significant and data analysis was carried out with IBM SPSS v26.0 (IBM Corp, Armonk, NY, USA).

## 3. Results

Five hundred and forty-nine patients were recruited from June 2016 to June 2018, covering a median follοw-up period since first diagnosis of 5 years. This cohort comprised all patients with available medical records who underwent either RAI or follow-up WBS after exogenous or endogenous TSH stimulation at the Nuclear Medicine Departments of the largest tertiary referral centers for DTC in Northern Greece. Seventy-six point one percent (*n* = 418) were women, with a mean age of 45.8 years (±14.52), while 11.3% were diagnosed with more than one histological type/subtype, and papillary type, both classical and follicular variants, was overwhelmingly present. RAI took place 132 (median IQR, 102–170) days post-surgery for 539 patients. Most of the patients were administered fixed empirical radioiodine activities, typically 3.7 GBq (100 mCi), (range: 30–200 mCi, depending on patients’ risk stratification and requirement of multiple radioiodine treatments). Biochemical and imaging results were recorded for two consecutive follow-up WBSs which were performed based on latest ATA guidelines [7], depending on patients’ evidence of disease and our clinical experience: 502 patients underwent one WBS after RAI and 59 with higher risk characteristics underwent a second WBS (18 of them underwent RAI more than once). Higher risk characteristics included clinicopathological features such as aggressive histology, advanced stage, invasiveness, and/or evidence of structural or biochemical disease. Less than 2% of the cohort underwent more than two follow-up WBSs (results not included). TSH levels were assessed for all patients at post-surgery scintigraphy, RAI, and follow-up WBSs and were determined to be over 30 mIU/L for all patients.

Table 1 and Table 2 present the main histopathological characteristics of all patients and referral surgeons’ score respectively.

Table 3 presents patients’ characteristics at the time of post-surgery scintigraphy, RAI, first and second follow-up WBS including biochemistry, dose and preparation administered, the time between surgery, and RAI.

RTT according to ATA GLs [7] for all patients is shown in Table 4, while the association between data from Table 1, Table 2 and Table 3 with those from Table 4 is presented in Table 5.

Table 5 correlations having a statistically significant *p*-value are as follows:

Patients with follicular malignant neoplasms had significantly higher evidence of a structural incomplete response (SIR) compared with other response categories (chi-square test, *p* = 0.001),

Patients with T1 stage were significantly more likely to show an excellent response (ER) than any other response category (chi-square test, *p* = 0.039),

None of the patients with distant metastases showed an ER. Patients without distant metastases were more likely to have an excellent RTT (chi-square test, *p* < 0.001).

**Figure 2:**
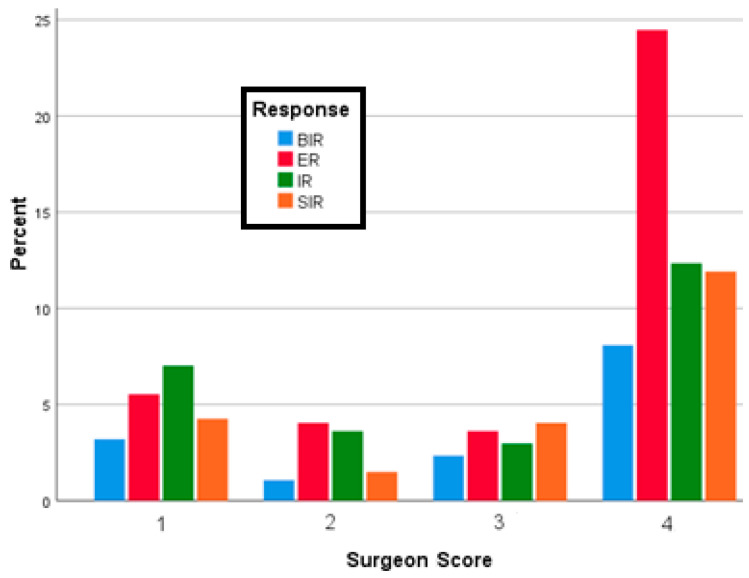
Surgeons’ score and RTT.

ER results were more prevalent in patients referred by a surgeon having a score of 4 than referred by less experienced surgeons (chi-square test, *p* = 0.037)—Figure 2.

During RAI the mean remnant score of ER is significantly lower than the incomplete response (IR) (*p* = 0.003) and SIR (*p* < 0.001), the mean remnant score of biochemical incomplete response (BIR) is significantly lower than SIR (*p* < 0.001), and the mean remnant score of IR is significantly lower than SIR (*p* < 0.001).

The same remnant score comparisons as point (v) are alsoverified for follow-up WBS, this time including the mean remnant score of BIR that is significantly lower than IR. Here *p* is always < 0.001.

During RAI the mean Tg value in ER, BIR and IR is significantly lower than SIR (*p* = 0.001 for all).

During follow-up WBS the mean Tg value in SIR is significantly higher than in ER, in IR and in BIR (*p* < 0.001 for all).

During follow-up WBS the mean Tg-Ab value in SIR is significantly higher than in ER (*p* < 0.001), in BIR (*p* = 0.028) and in IR (*p* = 0.042).

## 4. Discussion

Predictive staging systems are essential for accurate prognostic information, treatment algorithms, and exchange of accurate search information between medical centers. Although the prognosis of DTC is generally good, up to 10% of patients will eventually die due to the disease and an even greater proportion will face the morbidity of recurrences [8]. As a result, a number of studies have identified various clinicopathologic predictors for DTC and devised risk-group stratification or staging systems to identify those at high risk of death from cancer for more aggressive surgical and adjuvant treatment, while those at low risk would be spared aggressive treatment [9]. Despite the variability between these systems, the majority of prognostic factors were included in more than one of them and were therefore considered “widely accepted”. These include age, gender, tumor size and extension, LN involvement, and distant metastasis. Except for these widely accepted factors, in the current study we explored the association between recurrence and other prognostic factors suggested by additional literature such as Tg, Tg-Ab levels, TSH stimulation method, therapeutic activity administered, tumor multifocality, remnant size and intensity, as well as surgeons’ experience in effectively performing thyroidectomies. The assessment of these factors enables DRS performance, rather than a static evaluation of initial risk stratification. The predictive value of these factors and all the histopathological and clinical laboratory characteristics in the part of the sample examined highlights the paramount importance of DRS recurrence in patients with differentiated cancer, as already shown by two more studies [10,11].

Despite a higher incidence of DTC in females [12], as confirmed by our results, overall survival was found to be better in women [13]. It is hypothesized that worse outcomes in men may potentially be accounted for by a more aggressive behavior of DTC in these patients, and thus, male gender is considered as being an adverse prognostic factor. According to our results there is a slightly worse prognosis for men, but it is not statistically significant. One should consider gender-related ascertainment bias as a possible explanation for these findings. Perhaps men seek medical advice at an older age with a more advanced disease. Additionally, since women have lower all-cause mortality rates and live longer than men, gender-specific overall survival rates should be used instead of mortality. The impact of estrogen on thyroid cancer is debatable and numerous studies have reported conflicting results regarding hormone levels and progressive disease [14].

Advanced age is associated with increased mortality in many cancer types, yet TC is the only human malignancy to include age as part of the staging system [15]. Traditionally, an age cut-off of 45 and most recently of 55 years is used in current DTC staging guidelines. Although approximately half (52.3%) of our patients were over 45 years old, age was not confirmed by our statistical analysis as a prognostic factor. The use of an inaccurate cut-off point could explain this. Some studies found that the association between increasing age and worse outcome should be evaluated more accurately as a continuum, since no single point was found as an accurate cut-off point [16,17,18]. Clinically, this would mean that many patients with DTC would be down-staged, thus potentially avoiding unnecessary therapies.

Most studies agree that patients diagnosed with an FTC are more likely to succumb to their disease than those diagnosed with a PTC, with widely invasive and unspecified FTC conferring the least favorable prognosis. FTC tumors have a lower tumor differentiation and are more likely to be of higher TNM stage but these discrepancies do not explain completely the difference in prognosis [19]. Some variants of PTC have been associated with higher risk for disease recurrence and aggressiveness, while its follicular variant was identified as a significant protective factor [20].On the other hand, the follicular variant of PTC was identified as a significant protective factor for PTC recurrence. This is consistent with the behavior observed in several studies on follicular variant PTC that showed lower nodal metastases at presentation with similar long-term prognoses as those of classic PTC [21]. In concordance with these findings, patients with follicular neoplasms had significantly higher evidence of structural disease compared with other response categories (*p* = 0.001), while those with PTC or HC showed more probably ER and IR, and patients diagnosed with follicular variants of PTC were more likely to have ER.

DTC size is associated with poorer prognosis and mortality rates, as reports of patients with microcarcinomas (<1 cm) having extensive lymph node metastases remain rare. Given the current evidence, smaller size does not equal good outcomes. Primary tumors up to 1 cm in size are associated with a good prognosis, but they may have all the specific properties of malignant tumors, with nodal metastases, capsular infiltration, or multifocal growth in thyroid tissue [22,23]. Our results indeed revealed a correlation between stage and treatment response, since patients with tumors <2 cm (T1 (p)) showed ER in a larger percentage compared to patients for whom the histopathological report showed larger primary tumors (*p* = 0.039).

Multifocality is a common finding in DTC; however, its significance is controversial. In one multicenter study, multifocal thyroid cancer was associated with higher recurrence rates than unifocal disease; however, a difference in survival was not found to be significant [24]. In agreement with ambiguous results in the literature, we found no correlation between multifocality of the primary tumor and RTT. Recent studies suggest that associated poor prognosis is related to total tumor diameter rather than the number of tumor loci, which was not examined in the current study [25].

No or minimal extra-thyroidal extension (ETE) versus major ETE had a significant impact on DTC prognosis and some studies even found worse survival rates in patients with minimal extension rather than no ETE [26,27]. According to our results, patients with invasive primary tumors are not equally distributed (*p* = 0.003) in the groups of response. However, no statistically significant association was documented between invasiveness and RTT categories.

Nodal involvement and distant metastases are well-documented risk factors for recurrence in DTC [28,29,30]. Our cohort comprised 41.1% of patients with lymph node metastases, of whom 25.1% did not undergo lymph node neck dissection. There seems to be a correlation between nodal metastases and structural disease or indeterminate RTT, not statistically significant in our study, while the correlation between distant metastases and worse outcome is confirmed (*p* < 0.001).

Both surgeon experience and completeness of surgery are well-documented as critical to patient outcome [31] and a statistically significant correlation has been shown in our study as well, where only 8.5% of surgeons operated on 20 or more patients, while 76.6% of them operated on less than 5 patients. High remnant score for almost the whole cohort (92.2%) is revealing of the predominance of unexperienced surgeons performing thyroidectomies far from centers of excellence, reflecting the risks of managing DTC patients by non-highly-qualified physicians. A possible solution could be the concept of RAI as a backstop, when a suboptimal surgery exists as a possibility), since remnant score became 0 at follow-up WBS for 57.6% of the patients with only 4% having a remnant score ≥10. Remnant score was associated both at RAI and follow-up WBS with treatment response, showing a significant correlation between higher remnant scores and the presence of structural incomplete response.

Our findings, whichconfirm the importance of clinical data, suggest a new approach to risk stratification, considering not only an initial DTC stage, but also clinical features and response to treatment administered. Effectiveness of administered treatment regarding biochemical response, with the exception of the minority of patients who are at higher risk for recurrence and underwent a second WBS, is obvious for mean Tg and Tg-Ab concentrations (Concerning Tg, in accordance with the findings of other researchers [32,33,34,35], there was a statistically significant association of treatment response with both stimulated Tg at the time of RAI and follow up (*p* < 0.001). Stimulation method (rhTSH/THW) was not shown to interfere with recurrence rate, in agreement with Jeonghoon Ha et al., who showed that sTg levels using rhTSH at both RA and follow-up have a high negative predictive value and are as effective as using THW for predicting recurrence [36]. Tg-Ab interference with serum Tg measurements severely compromises the clinical utility of Tg monitoring of DTC patients for recurrence [37]. Concerning Tg-Ag, a statistically significant association with treatment response was observed only at follow-up. In case of high or rising Tg-ab levels, we recommend that the treating physician should actively try to diagnose recurrence, even if this remains rare for the majority of patients. Our data is in concordance with Verburg et al. [38], always keeping in mind that the trend is of greater value than the absolute level.

Despite the literature’s positive association of anti-TPOs with TC [38], association of pre-therapy anti-TPO levels with treatment response was not statistically significant in our study. We believe that our results are not in contradiction with published data, since most of them correlate the presence of positive anti-TPOs with the onset of TC and not with the prognosis of the disease.

## 5. Limitations

This is a retrospective study with considerable heterogeneity and gaps in the data recorded and analyzed. For example, thyroid remnant size was assessed using an unvalidated, partly subjective scoring system. Histopathological examinations were performed in different laboratories, which are not based in the tertiary centers examined. However, a central retrospective evaluation of histopathological assays was conducted by two experienced nuclear medicine physicians. Another gap in our data was ultrasonography (US) and FNA results, where accuracy is highly operator-dependent and were therefore not evaluated in the current study. Additionally, the study took place at tertiary referral centers in the European Union. Our results may thus not be generalizable to other geographic areas and may be conservative with respect to less specialized settings or more resource-constrained regions.

## 6. Conclusions

Most risk stratification systems include the same core parameters of age, tumor size, grade, presence of local invasion, and regional or distant metastases. According to our findings, treatment response had a statistically significant association with histology, T and M stages, invasiveness, surgeons’ experience, Tg levels, and remnant score both during RAI and follow up and Tg-Ab levels during follow-up WBS. A lack of definitive evidence continues to create confusion when conveying accurate prognostic information to the DTC patient population and when determining treatment regimen. In all cases a combined multidisciplinary approach, with consideration of the available guidelines and stratification systems, should be utilized when planning an individualized treatment program including follow up strategy, to offer the best contemporary care.

## Figures and Tables

**Table 1:** Histopathological characteristics.

Histological Type/Subtype % *n*	
Papillary, classical variant (P)	66.3% (364)
Papillary, follicular variant or mixed type (FVP)	29.5% (162)
Follicular (F)	8.9% (49)
Hurthle Cell (HC)	2.0% (11)
Insular Cell (IC)	0.7% (4)
Trabecular Variant Papillary (TVP)	1.8% (10)
Anaplastic (A)	0.9% (5)
Medullary Thyroid Cancer (MTC)	1.1% (6)
**TNM ^a^ Stage** **% *n***	
Τ1	53.9% (295)
T2	12.8% (70)
Τ3	31.4% (172)
T4	2.0% (11)
N0	33.9% (186)
Ν1	41.1% (225)
Nx	25.1% (138)
M0	94.7% (520)
M1	5.3% (29)
**Number of Lesions**	Mean (SD): 2.48 (2.52)
	Multifocality: 54.7% (299)
**Longest Diameter of Primary Tumor**	Mean (SD): 1.66 (1.16)
	>1 cm: 65.4% (359)
**Invasive (I) ^b^**	40.6% (222)
**Number of excised lymph nodes**	Mean (SD): 8.59 (12.01)
**Number of metastasized lymph nodes**	Mean (SD): 2.98 (6.03)

^a^ The histology report and the American Joint Committee on Cancer/Union Internationalecontre le Cancer:Tumour, Nodes, Metastasis (TNM) system, 6th edition, were used to stage each patient’s disease pre-ablation. ^b^ All primary tumors with vascular, minimal, or gross extrathyroidal extension were considered invasive.

**Table 2:** Referral Surgeons’ score.

Referrals	Median (IQR): 2.0 (1, 4)
Surgeons’ score (among 95 surgeons)	
1	76.84% (73)
2	8.42% (8)
3	6.32% (6)
4	8.42% (8)

**Table 3:** Diagnostic, treatment, and follow-up characteristics (median-extreme values or percentage-number of cases) at each time point.

Characteristic	Post-Surgery Scintigraphy ^a^ (*n* = 274)	RAI (*n* = 539)	1st WBS (*n* = 501)	2nd WBS (*n* = 55)
Tg-Ab, Median, Mean (IQR)	20.0, 65.78 (10.34, 32.30)	20.0, 58.35 (13.0, 34.12)	20.0, 52.47 (12.93, 27.0)	21.30, 55.37 (20.40)
TgAb > 30 IU/mL (*n*)	26.6(73)	28.6 (154)	21.6 (108)	30.9 (17)
Anti-TPO, Median, Mean (IQR) (*n* = 218)	20.85, 25.37 (11.43, 59.32)	-		
Anti-TPO > 30, % (*n*)	38.5% (84)	-		
TSH, Median, Mean (IQR)	95.48, 97.59 (60.90, 126.40)	120.0, 120.75 (85.0, 146.70)	108.0, 114.67 (83.46, 140.0)	116.50, 136.78 (89.0, 161.03)
Tg, Median, Mean (IQR)	2.09, 35.25 (0.82, 5.94)	2.10, 22.47 (0.69, 6.85)	0.44, 10.27 (0.30, 1.18)	3.50, 47.03 (0.62, 33.73)
Tg < 1, % (*n*)	30.5 (84)	33.2 (179)	73.5 (369)	37.3 (22)
Tg 1 < 5, % (*n*)	42.5 (117)	36.5 (197)	14.5 (73)	15.3 (9)
Tg 5–10, % (*n*)	9.1 (25)	10.8 (58)	4.0 (20)	10.2 (6)
Tg > 10, % (*n*)	17.8 (49)	19.5 (105)	8.0 (40)	37.3 (22)
Remnant score, Median (IQR), Mean (St.D.) (*n* = 218)	3 (2, 6), 4.37 (3.52)	6 (3, 8), 6 (4.53)	0 (0, 2), 1.94 (3.5)	3 (0, 9), 5.19 (5.2)
Stimulation method:				
THW	81.4% (223)	48.3% (260)	30.7% (154)	5.1% (3)
rh-TSH	18.6% (51)	51.7% (279)	69.3% (347)	94.9% (52)

^a^ Post-surgery scintigraphy was performed 6–8 weeks after thyroidectomy and 1 to 2 months before RAI.

**Table 4:** Global RTT.

Treatment Response Category	% (*n*)
ER (Excellent Response)	37.7% (177)
BIR (Biochemical Incomplete Response)	14.7% (69)
SIR (Structural Incomplete Response)	21.7% (102)
IR (Indeterminate Response)	26% (122)

**Table 5:** Characteristics and response to treatment.

			Response to Treatment			
			BIR	ER	IR	SIR
			*n* (%)	*n* (%)	*n* (%)	*n* (%)
**Main Characteristics**	Gender, *n* (%)	F	59 (16.5)	139 (38.9)	87 (24.4)	72 (20.2)
		M	10 (8.8)	38 (33.6)	35 (31)	30 (26.5)
	PTC, *n* (%)	No	23 (14.2)	56 (34.6)	39 (24.1)	44 (27.2)
		Yes	46 (14.9)	121 (39.3)	83 (26.9)	58 (18.8)
	Follicular variant of PTC, *n* (%)	No	48 (14.3)	118 (35.2)	96 (28.7)	73 (21.8)
		Yes	21 (15.6)	59 (43.7)	26 (19.3)	29 (21.5)
	F, *n* (%)	No	68 (16)	166 (39.1)	107 (25.2)	84 (19.8)
		Yes	1 (2.2)	11 (24.4)	15 (33.3)	18 (40)
	HC, *n* (%)	No	68 (14.8)	174 (37.8)	118 (25.7)	100 (21.7)
		Yes	1 (10)	3 (30)	4 (40)	2 (20)
	Insular, *n* (%)	No	68 (14.6)	177 (38)	120 (25.8)	101 (21.7)
		Yes	1 (25)	0 (0)	2 (50)	1 (25)
	TVP, *n* (%)	No	68 (14.7)	174 (37.7)	122 (26.4)	98 (21.2)
		Yes	1 (12.5)	3 (37.5)	0 (0)	4 (50)
	A, *n* (%)	No	69 (14.8)	176 (37.7)	121 (25.9)	101 (21.6)
		Yes	0 (0)	1 (33.3)	1 (33.3)	1 (33.3)
	MTC, *n* (%)	No	45 (14.4)	139 (44.6)	65 (20.8)	63 (20.2)
		Yes	2 (33.3)	2 (33.3)	1 (16.7)	1 (16.7)
	StatusN, *n* (%)	N0	29 (18.4)	64 (40.5)	35 (22.2)	30 (19)
		N1	21 (11)	72 (37.7)	50 (26.2)	48 (25.1)
		Nx	19 (15.8)	40 (33.3)	37 (30.8)	24 (20)
	StatusM, *n* (%)	M0	67 (15.1)	177 (39.9)	118 (26.6)	82 (18.5)
		M1	2 (7.7)	0 (0)	4 (15.4)	20 (76.9)
		Μ0	0 (0)	0 (0)	0 (0)	0 (0)
	StageT_new, *n* (%)	Any T1	40 (16.1)	107 (43.1)	52 (21)	49 (19.8)
		T2	10 (18.2)	17 (30.9)	14 (25.5)	14 (25.5)
		Any T3	19 (11.9)	52 (32.7)	53 (33.3)	35 (22)
		Any T4	0 (0)	1 (12.5)	3 (37.5)	4 (50)
	Stimulation method, *n* (%)	rhTSH	25 (21.2)	42 (35.6)	30 (25.4)	21 (17.8)
		THW	23 (10.7)	79 (36.9)	64 (29.9)	48 (22.4)
	Age at diagnosis mean (St. D.)		46.86 (14.5)	46.18 (14.43)	46.63 (14.26)	45.3 (14.55)
	Multifocality, % (*n*)		56.5 (39)	54.5 (96)	48.4 (59)	63.7 (65)
	Invasiveness % (*n*)		14.7 (28)	33.0 (63)	28.8 (55)	23.6 (45)
**Surgeons’score, % (*n*)**	1		21.7 (15)	14.7 (26)	27 (33)	19.6 (20)
	2		7.2 (5)	10.7 (19)	13.9 (17)	6.9 (7)
	3		15.9 (11)	9.6 (17)	11.5 (14)	18.6 (19)
	4		55.1 (38)	65 (115)	47.5 (58)	54.9 (56)
**RAI**	Activity administrated, mean (St.d)		96.30 (16.13)	96.05 (11.29)	95.57 (13.85)	97.84 (19.68)
	remnant_score, mean (St.d)		4.16 (3.08)	3.08 (1.93)	4.55 (3.19)	7.09 (4.79)
	Tg, mean (St.d)		3.47 (3.87)	2.86 (4.05)	17.47 (87.77)	141.37 (369.66)
	Tg-Ab, mean (St.d)		28.28 (29.83)	39.38 (69.54)	53.89 (214.17)	155.87 (569.77)
	TM, median (IQR)		27.10 (9.81, 199.30)	19.50 (13.56, 59.81)	21.30 (9.93, 56.32)	19.00 (11.31, 58.90)
**WBS**	remnant_score, mean (St.d)		0.58 (0.34)	0.74 (0.45)	2.8 (2.95)	5.6 (5.1)
	Tg, mean (St.d)		1.15 (4.48)	0.52 (0.79)	1.8 (2.98)	49.15 (152.42)
	Tg-Ab, mean (St.d)		42.06 (32.71)	20.96 (20.04)	57.98 (183.67)	119.84 (439.81)

## Supplementary Materials

The following are available online at https://www.mdpi.com/2077-0383/9/9/2708/s1, Table S1: Assay methodology, Table S2: Reference values for TgAb.
