# Supplementary material for: Dynamic Risk Stratification for Predicting Treatment Response in Differentiated Thyroid Cancer
Source: J Clin Med. 2020 Aug 21;9(9):2708. doi: 10.3390/jcm9092708 (PMC7565378; doi:10.3390/jcm9092708)
Supplement: Supplementary file 1 [file jcm-09-02708-s001.pdf]

Annex

Table S1. Assay methodology

Analyte	Name (Manufacturer, location)	FAS or LLD	Intra-assayCV [a]	Inter-assayCV [a]
Tg [b]				
PGH	B·R·A·H·M·S Tg-pluS RIA, Thermo Scientific Biomarkers, Hennigsdorf, Germany	0.3 μg/L [c]	1.0%–13.9%	1.1%–21.0%
CHT	B·R·A·H·M·S TgS RIA, Thermo Scientific Biomarkers, Hennigsdorf, Germany	0.1 μg/L [c]	0.9%–2.9%	2.2%–3.6%
TgAb [c]				
PGH	B·R·A·H·M·S Tn RIA, Thermo Scientific Biomarkers, Hennigsdorf, Germany	0.1 IU/mL	2.0%–7.5%	3.1%–5.5%
CHT	Dia Source TG AB, RIA R-CI-100,Louvain-la-Neuv e – Belgium	6 IU/mL	1.6%–4.9%	2.1%–6.3%
Anti-TPO				
PGH	B·R·A·H·M·S anti-TPOn Thermo Scientific Biomarkers, Hennigsdorf, Germany	5.0 IU/mL	1.9%–4.8%	3.7%–9.1%
CHT	Dia Source TPO AB, RIA R-CO-100,Louvain-la-Neu ve – Belgium	1.3 IU/mL	2.7%–5.5%	4.2%–9.5%

PGH, Papageorgiou Hospital, *CHT*, Cancer Hospital Theagenio, Anti-TPO, anti-thyroperoxidase autoantibodies; CV, coefficient of variation; FAS, functional assay sensitivity; LLD, lower limit of detection; Tg, thyroglobulin; TgAb, anti-thyroglobulin autoantibodies. [a] CVs depended on analyte level or antibody titre.[b] Tg assays were calibrated to the Certified Reference Material-457 standard.[c] The interfering cut-off of TgAb was 20IU/ml

Table S2. Reference values for TgAb [a]

TgAb value	Interpretation
>30 IU/ml	Negative for Thyroglobulin autoantibodies
30–70 IU/ml	Limit
>70 IU/ml	Positive for Thyroglobulin autoantibodies

[a] Values reported above are indicated by the manufacturer who recommends that each laboratory determines its own reference interval.
